# Supplementary material for: A qualitative study of health visitors’ family focused practice with mothers with mental illness in Northern Ireland: Perspectives of health visitors, mothers and partners
Source: PLoS One. 2024 Aug 15;19(8):e0306890. doi: 10.1371/journal.pone.0306890 (PMC11326591; doi:10.1371/journal.pone.0306890)
Supplement: Attachment — Submitted filename: Response to reviewers (final).docx [file pone.0306890.s001.docx]

| **Academic Editor’s comments** | **Response** |
| --- | --- |
| Please ensure that your manuscript meets PLOS ONE's style requirements, including those for file naming | We have checked requirements and understand that our paper meets them. |
| We note that you have referenced Bunting L, McCartan C, Davidson G, Grant A, McBride O, Mulholland C, et al. which has currently not yet been accepted for publication. Please remove this from your References and amend this to state in the body of your manuscript: (Bunting L, McCartan C, Davidson G, Grant A, McBride O, Mulholland C, et al. [Unpublished]) | We have removed this reference completely. |
| The title and/or the abstract should reflect where the study was carried out (country and region). | The title has been amended as follows:  A qualitative study of health visitors’ family focused practice with mothers with mental illness in Northern Ireland: perspectives of health visitors, mothers and partners |
| - The design and method section indicates that this paper is based on the qualitative findings of a larger mixed methods study. However, it doesn’t say in details about the study approach of the qualitative part. Is it just a simple descriptive or analytical qualitative study or based on any theoretical approach? | The second (qualitative) phase employed a descriptive qualitative design and the information below was added into the last paragraph on page 5 as follows:  The second phase employed a qualitative descriptive design. Individual semi-structured interviews were undertaken with 10 health visitors who obtained the highest and lowest FFP scores on the FFMHPQ to further explore and expand upon findings (lowest score = 59, highest score = 140). |
| - Sampling and data collection techniques are clear and specific. Better to write the average time (as stated 60 minutes) with minimum time and maximum time of the interview. I don't suggest using the term 'generally lasted'. | The following information has been provided into page 10 as follows:  Interview duration ranged from, 31 – 62 minutes for mothers, 30 – 46 minutes for partners, and 37 – 70 minutes for health visitors. |
| - In the first para of 'discussion' section, it states "findings highlighted low level of FFP". This needs to be elaborated as the findings are qualitative and it's hard to say the low level in specific. It would be better if you provide some additional information in the data analysis section to support this claim. Since the low levels of FFP is one of the conclusions of the study, I suggest to make it clearer and more specific. | The reference to ‘Low levels of FFP’ has been replaced in the discussion and conclusion with ‘restricted scope’, as follows:  Discussion  Findings highlighted the restricted scope of health visitor’s FFP.  Conclusion  This study found that the scope of health visitors’ FFP is restricted as they focus predominantly on mothers and children instead of also supporting partners. |
| - I also suggest to illustrate the themes and relevant codes in a diagram as diagrammatic illustrations are very beneficial for the audience/readers to get a glimpse of the findings and guide them thoroughly in understanding the findings/discussion | We have now added Table 5: themes and sub themes |
| If there are restrictions on publicly sharing data—e.g. participant privacy or use of data from a third party—those must be specified. | We cannot make the raw data accessible/published. This is because:  We had informed participants of this study that data would be used within publications and presentations. However, while we are in support of the open science framework and are also committed to ethical data sharing we did not include any detail regarding data repositories within participant information. |
| **Reviewer one comments** |  |
| In the final sentence of the conclusion, it is mentioned that all the health visitors in the study exceeded the recommended workloads. I feel like this information should have been mentioned earlier as it provides important context for the findings. | We have now included reference to this important point within the first paragraph in the discussion. |
| *Title*  Should it say ‘partners’ rather than spouses? | The title has been revised and the term spouse has been replaced with partner |
| *Abstract*  I thought that the abstract was very comprehensive but could perhaps highlight the shortage of resources as mentioned above. | We have revised the abstract. The conclusion within abstract now makes reference for need for health visitors to receive necessary training and time to engage in FFP. |
| *Introduction*  Page 4. A brief explanation of psycho education would be helpful.  Final sentence only mentions mothers and partners but the interview questions (on page 9) suggest that health visitors were also asked about the adequacy of support. | A brief explanation of psycho education has now been provided in page 4 as follows:  Psycho education entails providing written, verbal, and/or online information to facilitate mothers, partners and children to understand mental illness and how to cope with it (Foster et al. 2018).  Th final sentence on page 4 has been refined as follows:  It also aimed to explore the acceptability and adequacy of this support from health visitors, mothers and partners’ perspectives. |
| *Design and methods.* I’m not sure whether the description of the survey stage of the study needs to be detailed here, but that is for the editors to advise. The authors might just need to refer to the survey results as providing criteria for selecting the sample of health visitors.  Page 6. The authors assigned pseudonyms to the participants but did not use them when reporting the findings. I wondered why this might be? | We would like to leave this as the primary focus of the paper is the qualitative results and we have included a paragraph on page 5 about the broad focus of the survey and its psychometric properties and referenced our published EFA paper and the study protocol for further detail. On page 5 we also indicated that that those who obtained the highest and lowest scores on the survey were recruited and defined what the lowest and highest scores were.  We have prefixed the quotes with various salient demographic details, including service type, whether respondent was a mother or partner or professional and age of mother so we felt that to also use pseudonmys would have made the text more difficult to read and would not have added anything substantial. |
| *Data collection* Page 7, penultimate sentence says ‘both’ [perspectives] which implies 2 rather than the 3 that were gathered.  Page 8. Only the topic guide for the health visitor interviews is included. It would be helpful to see the topic guide(s) used in mothers’ and partners’ interviews. | The word both has been removed.  These have now been included along with the following text on page 8:  For additional detail regarding topic guides see Table 1 for health visitors, Table 2 for mothers and Table 3 for partners. |
| *Findings* Research participants. Page 12. It would be helpful to know how many of the health visitor participants were trained in FFP. | While we measured professionals’ training in FFP in phase one we did not collect this information in phase two. |
| *Themes* Pages 15 – 17. It would be easier to follow if some of the longer quotes were indented. There are also some long complex sentences which might be better broken up (e.g. the sentence beginning ‘Relatedly’ on page 16).   Page 21. This section highlights resource issues relating to training (when is mental illness beyond their professional boundaries?) and workload. It was interesting that health visitors also alluded to their own needs (e.g. ‘mentally drained’) and I wondered if/how these were being met. This could be picked up in the discussion.  Page 23. This section highlights how health visitors are able to empathise with the women in their care. It made me wonder whether recommendations to employ more male health visitors were appropriate. | Sentences on page 15 – 17 have been refined to enhance readability.  Reference to excessive caseload and also to health visitors feeling mentally drained has now been referred to in first para in discussion and discussed.  We would like to retain this recommendation because having greater numbers of male health visitors may encourage more health visitors to engage fathers as well as help fathers to engage with the service. In the discussion we highlight that health visitors are primarily female and this has perpetuated the feminine nature of care provided within health visiting (36), and secondly perpetuates father’s perceptions that the service is designed for mothers (18). A global review of services has called for a more gender-balanced healthcare workforce within all professional categories (37). |
| *Discussion* P24. I realise I keep mentioning this, but I think that the issue of workload should be mentioned in the first paragraph. It might explain why health visitors prioritised the needs of mothers rather than partners’ needs.    Fathers as financial providers is a bit of a gender stereotype – it needs to be clearer that that is their perception of themselves (if indeed that was the case for all partner participants).  Overall, I think that the manuscript is very well-presented, and I do not wish my suggestions to be taken as unduly critical. The authors are free to disregard my alternative interpretations of the findings which come from my own research with healthcare professionals who often struggle to provide the level of care that they would like to within time/resource constraints. | Thanks for your careful review which we appreciate as we feel you have picked up important points that we have missed, such as caseload, which do merit adequate discussion. We have now included this into discussion in the first paragraph as detailed below:  Supporting mothers’ mental health took a toll on the health visitors themselves, with health visitors often feeling mentally drained. Health visitors provided this support in the context of excessive workloads and high caseloads of families. This may partly explain their restricted scope of practice with families impacted by maternal mental illness. Reid and Tracey’s (2023) integrative review, exploring health visitors’ workload, also highlighted how excessive caseloads can negatively impact the service they provide.  Thanks for alerting us to this. We have now included a quote that illustrates how partners want to be the provider and protector but we have removed reference to financial provider as this was not their own perception.  Thanks for your careful review which we appreciate as we feel you have picked up important points that we have missed, such as caseload, which do merit adequate discussion as they impact health visitors’ capacity. We have now included this into discussion in the first paragraph. |
